# A catalog of automated analysis methods for enterprise models

**DOI:** 10.1186/s40064-016-2032-9

**Published:** 2016-04-02

**Authors:** Hector Florez, Mario Sánchez, Jorge Villalobos

**Affiliations:** Department of Systems and Computing Engineering, Universidad de los Andes, Bogotá, Colombia

**Keywords:** Enterprise analysis, Enterprise models, Enterprise architecture, Quantitative analysis, Functional analysis

## Abstract

Enterprise models are created for documenting and communicating the structure and state of Business and Information Technologies elements of an enterprise. After models are completed, they are mainly used to support analysis. Model analysis is an activity typically based on human skills and due to the size and complexity of the models, this process can be complicated and omissions or miscalculations are very likely. This situation has fostered the research of automated analysis methods, for supporting analysts in enterprise analysis processes. By reviewing the literature, we found several analysis methods; nevertheless, they are based on specific situations and different metamodels; then, some analysis methods might not be applicable to all enterprise models. This paper presents the work of compilation (literature review), classification, structuring, and characterization of automated analysis methods for enterprise models, expressing them in a standardized modeling language. In addition, we have implemented the analysis methods in our modeling tool.

## Background

Enterprise architecture (EA) encompasses different kinds of elements such as principles, methods, and models, which are used by domain experts in order to provide a holistic view of an enterprise. EA projects are supported on the construction of models that abstract the enterprise for understanding its organizational and technological aspects (Lankhorst 2005); typically, these models are focused on structural aspects of the enterprise, and serve for documentation, communication, diagnosis, analysis, discussion, and design purposes. Enterprise modeling (EM) is the process of creating an integrated enterprise model, which represents certain aspects of the enterprise that are required for the modeling purpose. An enterprise model describes the current or future state of an enterprise and contains the enterprise knowledge of the stakeholders involved in the modeling process (Sandkuhl et al. 2014). Enterprise modeling offers different views of an enterprise, for understanding enterprise systems through proper abstractions (Frank 2014).

When an enterprise model is used to analyze the enterprise, analysis results can be used to support decision making processes, such as planning future states of the enterprise (Buckl et al. 2009); as a result, analysis has become a critical task because it contributes to the improvement of the business and IT elements. Analysis is a complex human activity that involves the formulation of hypotheses and the discovery of insights in order to get assessments. Typically, one analyst manipulates the enterprise model in order to extract information that is useful, for evaluating the state of the enterprise. Nevertheless, the quality of the analysis results depends on three factors: (1) the experience, knowledge, and skills of the analyst; (2) the quality of the models; and (3) the granularity, completeness and level of detail of the information contained in the models. The implementation of automated analysis methods contributes to reduce the risk associated with the first factor.

Automated analysis methods are algorithms that extract information from the model and make calculations. Automating the procedures for extracting and calculating information makes possible to work with the complete model, which can include a large amount of elements and relations. Automated analysis methods might require that the model has some specific information; thus, the metamodel of the modeling language (e.g., ArchiMate) must be adapted in order to demand the required information in the model. The results provided by one analysis method not only are used to provide assessments, but also could be used to enrich the model, for running more complex analysis methods.

In the literature, there are several analysis methods for different purposes (e.g., optimization, performance, and impact of change); however, normally those analysis methods are focused on a particular problem and they are based on different metamodels (e.g., workload of human resources in business processes using BPMN as modeling language, for a commercial enterprise). Then, the analysis method algorithm might be very specialized making it neither applicable nor reusable in other enterprise models. The lack of re-usability becomes an important issue because, when the analyst decides to perform an existing analysis method that was not designed for the enterprise model that is being analyzed, there is the need to modify the analysis method increasing the effort and cost of the analysis process. Moreover, due to analysis methods are taken from multiple references; there is a lack of uniformity, structure, and characterization.

In this work, we have made the compilation, classification, structuring, characterization, and unification of the analysis methods found in the literature review. The result of this work has been collected in one catalog of analysis methods to offer, for each analysis method, a unified characterization, detailed description, required inputs, algorithm, and results. This catalog is also extensible in order to allow the inclusion of new analysis methods. The catalog should serve several purposes. For example, it should assists analysts in the identification of proper analysis methods considering the goals and characteristics of the enterprise that is being analyzed. The catalog also create requirements for modelers, whose goal is to create models that are complete with respect to specific business objectives. Furthermore, the catalog supports stakeholders in identifying certain aspects of the enterprise that matches their concerns.

Although the catalog is intended to be independent of the modeling language, we faced the necessity of selecting a coherent and well-known set of concepts in order to describe the analysis methods. For this purpose we selected ArchiMate, a standardized and well known language based on TOGAF’s Architecture Content Framework, which was created around the most commonly concepts used in EA. The modeling tool that we have created to implement, experiment and validate the analysis methods found in the catalog is thus based on ArchiMate. By means of this tool we were able to identify specific features for each analysis method, determine the requirements for their execution, design and test the algorithms for each one, and ultimately document them to form the catalog. Moreover, the fact that ArchiMate is founded on generic and common concepts, makes the analysis methods concrete but also as general as they can be. Furthermore, the tool allows adjusting, adapting, or personalizing the ArchiMate metamodel to (1) ensure that the model contains the required information for performing the desired analysis and (2) enrich the model with the analysis methods results. In addition, when the catalog will evolve to including new analysis methods, these will be easily implemented and validated thanks to its extensible architecture. For illustrating the analysis methods of the catalog, we have built an enterprise scenario, which is one publisher of academic books. The enterprise model of this scenario has been built using the tool that we have created; thus, this model is based on ArchiMate as modeling language. It has 184 business elements, 13 application elements, 13 infrastructure elements, 28 motivation elements, and 432 relations arranged in 12 views. This scenario is proper for validating this work because it has several issues that can be solved through performing the analysis methods of the catalog.

The rest of the paper is structured as follows. The next section summarizes concepts regarding enterprise analysis. In the third section, we present the systematic literature review made regarding enterprise analysis. In the fourth section we (1) classify and characterize the analysis methods taken from the literature review, (2) collect them in one catalog of analysis methods for enterprise architecture models based on the ArchiMate modeling language, and (3) illustrate some analysis methods using the experimental enterprise scenario. Then, in the fifth section we present our tool in which we have implemented the analysis methods of the proposed catalog. Finally, the last section concludes the paper.

## Enterprise analysis

Enterprise analysis is the application of property assessment criteria on enterprise models (Johnson et al. 2007). This means that, given an enterprise property and a criterion for assessing that property, doing model analysis requires evaluating said criteria using the information available in the model. Typically, model analyses are performed by humans supported by modeling tools that are used just to get access to the available data in an efficient way. However, analysts have the entire responsibility of discovering information (Florez et al. 2014) useful to provide assessments. On the one hand, this can be an advantage because humans are good at reasoning with incomplete information, but on the other hand, this can be a serious problem because the omission of elements and miscalculation of results, especially in large enterprise models, are very likely.

When the analyst performs an analysis using a modeling tool that is not conditioned for analysis processes, based on the purpose of the analysis, the analyst determines which elements, relations, and attributes are involved. The analyst browses the model using a modeling tool and gets the desired information from the model, in order to obtain the required results that can be included manually in the model. Those results are interpreted by the analyst for providing assessments.

### Automated analysis

Automated analysis refers to the use of automated analysis methods, for supporting analysts in the analysis process. Automated analysis methods are algorithms that allow extracting and calculating information from the model, for (1) obtaining facts that are results based on the information placed in the model or (2) enriching the model augmenting it with elements, relations, elements’ attributes, or relations’ attributes in order to provide new useful information.

Analysis methods require that the enterprise model provides sufficient and adequate information; then, the model must have specific required information in its elements or relations that are object of the analysis. Thus, when the purpose of building an enterprise model is analyzing the enterprise, modelers need to know the analysis requirements in order to create the necessary elements, attributes, and relations in the model. The lack of certain elements in the model could result in the impossibility to perform certain analysis methods. For instance, application components depend on infrastructure elements such as devices, system software, and infrastructure services. The availability of each infrastructure element should be 100 %; however, in the reality, there are a lot of factors that affect this requirement. Then, the availability of application components must be calculated based on the availability of the related infrastructure elements. Thus, one analysis method, for calculating the availability of application components, requires that the infrastructure elements in the model include their availability. Then, the modeling tool should be able to support and validate this characteristic.

For performing analyses, the analyst executes one analysis method using an *Analysis Engine* that works on the model and some input parameters provided by the analyst. After executing the analysis method, the *Analysis Engine* generates results, which are interpreted by the analyst, for communicating assessments.

#### Analysis dimensions

Lankhorst (2005) classifies enterprise architecture (EA) analysis approaches using four dimensions represented in a Cartesian plane. On the one hand, the Y-axis makes the distinction between quantitative and functional analysis. On the other hand, the X-axis makes the distinction between simulation and analytical techniques.

Quantitative analysis provides results regarding specific measures of the enterprise model. This analysis serve for several purposes such as optimization (e.g., quantification of the effect of design choices), impact of change (e.g., quantitative effect of changes), or capacity planning (e.g., amount of people required to finish processes on time). Enterprise models can be quantified by different measures such as performance measures that are time-related measures (e.g., response times, throughput), reliability measures (e.g., availability, dependability), or cost measures (e.g., architecture cost, ROI).

Functional analysis provides results that refer to information from functional aspects, which can be static or structural (e.g., structural properties) and dynamic or behavioral (e.g., services or activities). The analysis of static structure focuses on the syntactic i.e., symbolic representation of the elements and its relations. In one EA model, these analyses focus the separation of concerns, which allows managing the complexity of the architecture. The analysis of the dynamics focuses on the formal semantics, and is based on formal approaches such as process algebras and flow networks. Thus, a functional behavior analysis based on formal methods is a qualitative analysis that can detect logical errors, leads to a better consistency, and focuses on the logic of models.

## Analysis literature review

In order to analyze enterprise models, we performed a Systematic Literature Review (SLR), for identifying analysis methods suitable with the needs of enterprises. Based on Lankhorst (2005) classification, we have selected references, which propose approaches suitable with the quantitative and functional analysis dimensions. The method of the SLR includes research questions, search process, and inclusion exclusion criteria (Kitchenham et al. 2009, 2010; Lisboa et al. 2010). The research questions addressed in this work are:

- RQ1. What enterprise automated analyses have been addressed? Enterprise automated analysis are performed based on models, but models are created in different modeling languages. We are interested in finding automated analysis addressed by different authors in the enterprise context. In order to address RQ1, we identified automated analyses published in journals, conferences, and books.
- RQ2. What enterprise automated analyses are proposed by enterprise frameworks? Enterprise frameworks provide elements such as definitions, methods, guidelines, techniques. We consider that based on these elements, it is possible to assert some automated analysis that have not been addressed.
- RQ3. What analyses of models and graphs have application in the enterprise context? Since automated analysis are based on models, we are interested in including graph analyses (e.g., topological analysis) and models analyses (e.g., conformance analysis) in order to discover enterprise insights based on the characteristics of the model.

The search process was a manual search of conference proceedings, journals, and books that include studies regarding enterprise, graphs, or models analysis, regardless of impact factors and other bibliometrics. This search was done by consulting scientific literature databases such as *IEEE Explore*, *ACM Digital Library*, *Springer*, and *Oxford Journals*, among others; however, we also searched specific enterprise modeling journals that includes works about enterprise analysis. Selected conferences proceedings, journals, books, and frameworks are presented in Table 1. Selected conference proceedings have program committees and program chairs well known in the field. In addition, the search process included enterprise frameworks which propose enterprise analyses which might be automated. Ultimately, among those frameworks we only included TOGAF because it compiles elements included in previous models and because it is, among the most popular frameworks, the one that delves the most into methodological aspects.

**Table 1:** Selected journals, books, and conference proceedings

Source	Type
ACM/IEEE International Conference on Software Engineering	Conference Proceedings
ACM/IEEE International Symposium on Microarchitecture	Conference Proceedings
Enterprise Modelling and Information Systems Architectures	Journal
European Conference on Software Architecture	Conference Proceedings
Graph theory	Book
High availability and disaster recovery: concepts, design, implementation	Book
IEEE International Enterprise Distributed Object Computing Conference	Conference Proceedings
IEEE Workshop on Software Development Governance	Conference Proceedings
International CMG Conference	Conference Proceedings
Interoperability of Enterprise Software and Applications	Journal
IT professional	Journal
Journal of Information, Law & Technology	Journal
Journal of Object Technology	Journal
Long Range Planning	Journal
Model-Driven Engineering Languages and Systems	Book
Network design for IP convergence	Book
Sloan management review	Journal
Software quality attributes and trade-offs	Book
TOGAF Version 9.1	Framework

Once literature in enterprise analysis was collected, we had to asses whether or not it was relevant. To achieve this, we defined inclusion and exclusion criteria. The inclusion criteria were:

- Peer reviewed articles or books, which propose enterprise analysis techniques that can be classified in the quantitative or functional dimension.
- Peer reviewed articles or books, which propose graph or model analysis.
- Enterprise frameworks that provide enterprise analyses.

The exclusion criteria were:

- Studies that propose enterprise analysis techniques focused on simulation, animation, or another technique that illustrate dynamic behavior of the enterprise.

Based on the search process and on the inclusion and exclusion criteria, a set of references were selected. Selected references are presented below grouped by: quantitative dimension, functional dimension, or both dimensions (i.e., references that provide analyses applicable in quantitative and functional dimensions).

Regarding quantitative dimension, Iacob and Jonkers (2006) present and approach, for the quantification of performance of enterprise architectures applicable in the business, application, and infrastructure layers of ArchiMate. Davenport and Short (1990) propose a five-step approach for redesigning processes with IT in order to optimize the efficiency of the processes in enterprises. Armistead et al. (1999) expose a strategic view of business process management, useful for measuring business process effectiveness and human resources influence in business processes. Akkary and Driscoll (1998) present an architecture that improves instruction supply based on infrastructure resources such as memory and processor. Menascé and Bennani (2006) present analytical models for a wide range of multithreaded software server architectures. Di Penta et al. (2010) motivate the problem of analyzing the evolution of licensing statements and propose an approach to automatically track the licensing evolution of systems, identifying changes in licenses and copyright years. Somasundaram and Shrivastava (2009) explain information storage techniques, for managing and analysing storage volumetrics. Quartel et al. (2010) present a valuation approach to determine the cost and contribution of IT to the business. Berander et al. (2005) present management-oriented quality attributes models, which allows analysing the relation between quality attributes in IT elements. These works contribute with analyses that provide results based on specific indicators (e.g., availability, performance, optimization).

Regarding functional dimension, Sunkle et al. (2013) propose an approach to demonstrate how to perform impact of change analyses in the EA context based on ontologies. In The Open Group (2012), ArchiMate is presented by The Open Group as and EA modeling language, where structure and behavior is defined. Guadamuz (2001) explains *Habeas Data* as a legal tool for data protection providing elements to analyze the way in which IT comply data security. Lam (2002) presents an approach to business continuity planning (BCP) in order to avoid falls based on a practical value in a wide range of IT-related organizations. Angelov et al. (2009) study several reference architectures in order to investigate the contextual factors that influence the success of a reference architecture. Gómez et al. (2014) propose an approach for managing model and metamodel conformance, for the EA context making the distinction between linguistic and ontological conformance. In The Open Group (2011), TOGAF is presented by The Open Group as and EA standar framework. Bollobás (1982) presents the graph theory that is suitable for topology analyses. Kofman et al. (2009) present a governance solution for specifying governance capabilities, decision rights and responsibilities. Fischer et al. (2007) propose a federal approach for EA maintenance, which involves the participation of business actors and roles. These works provide analysis methods, for determining certain characteristics of the architecture (e.g., alignment, coherence) or the model (Graph structure, conformance).

In addition, there are references that include analysis in both, the quantitative and the functional dimensions. Donoso (2009) addresses network design providing strategies for managing and analyzing network resources and volumetric. Schmidt (2006) presents solutions for planning and implementing successfully high availability and disaster recovery providing needed server requirements to support business operation. Florez et al. (2014) present a proposal for analysing enterprise models based on ArchiMate modeling language, illustrating availability and requirements of infrastructure and application layers.

The literature review allowed us not only to identify analysis methods, but to understand the diversity of enterprise analyses. This diversity helped us to classify analysis methods in a more specialized category than the analysis dimension.

## A catalog of automated analysis methods

There are a lot of enterprise analyses that can be performed automatically based on an enterprise model. In order to propose a catalog of a1utomated analysis methods, we have made the following activities: (1) literature review (presented in the previous section) regarding analysis; (2) identification of analysis methods based on the literature review; (3) classification of identified analysis methods by analysis dimension (quantitative and functional); (4) classification of identified analysis methods by analysis type; (5) characterization of analysis methods; (6) unified documentation of each analysis method; (7) development of the algorithms, for each analysis method; (8) implementation of the analysis methods. The catalog then contains valuable information of each analysis method that can be used by analysts and modelers as a guide, when they are interested in creating enterprise models for analyzing the enterprise. On the one hand, when the analyst wants to analyze the enterprise, it is important to know what kinds of analyses are going to be performed. Then, the analyst can use the catalog in order to identify which automated analysis methods provide proper results. On the other hand, the catalog can be used by the modeler to know which information is mandatory, for performing desired analysis methods.

Based on the characteristics of the identified analysis methods, we proposed the following 16 analysis types: Performance (Pe): measures the ability of the system, for complying its activities based on a desired parameter (e.g., response time); Optimization (Op): estimates the efficiency and effectiveness of the system processes; Impact of Change (IoC): determines the consequences of modifying the system; Capacity Planning (CP): estimates needed resources, for achieving desired goals; Cost (Cos): establishes costs of the business; Availability (Av): estimates the percentage of time that the system is working correctly; Trade-Off (TO): measures the relation between quality attributes; Human Resources (HR): provides different kinds of results regarding actors or roles; Alignment (Al): determines if elements of different layers of the system are correctly adjusted; Coherence (Coh): verifies that all elements of the system have proper related elements; Correctness (Cor): corroborates that the system complies the requirements; Conformance (Con): finds out errors regarding the relation between the model and the metamodel of the modeling language; Gap (Ga): presents the differences between AS-IS and TO-BE models; Graph Structure (GS): checks the topology characteristics of the model; Count (Cou): calculates the amount of elements or relations of the model based on a given parameter; and Process (Pr): finds out specific relations between business processes and another desired elements of the model. The 16 analysis types were proposed in order to classify the analysis methods in a more specific way than just the analysis dimension. The types were taken based on the purpose of the identified analysis methods.

We have identified 78 analysis methods as result of the SLR. These methods were selected because they can be addressed in the enterprise context and they can be implemented to be performed automatically. Table 2 presents the distribution of the references of the literature review made in this work, sorting the references by dimension and type.

**Table 2:** Distribution of references

Dimension	Type	References
Quantitative	Pe	Iacob and Jonkers (2006)
	Op	Armistead et al. (1999) and Davenport and Short (1990)
	Ioc	Akkary and Driscoll (1998), Donoso (2009), Menascé and Bennani (2006) and Di Penta et al. (2010)
	CP	Donoso (2009), Schmidt (2006) and Somasundaram and Shrivastava (2009)
	Cos	Quartel et al. (2010)
	Av	Florez et al. (2014) and Schmidt (2006)
	TO	Berander et al. (2005)
	HR	Armistead et al. (1999) and Davenport and Short (1990)
Functional	IoC	Sunkle et al. (2013)
	Al	The Open Group (2012)
	Coh	The Open Group (2012)
	Cor	Donoso (2009), Florez et al. (2014), Guadamuz (2001), Lam (2002), Schmidt (2006) and The Open Group (2012)
	Con	Angelov et al. (2009) and Gómez et al. (2014)
	Ga	The Open Group (2011)
	GS	Bollobás (1982) and The Open Group (2012)
	Cou	The Open Group (2012)
	Pr	Angelov et al. (2009) and Kofman et al. (2009)
	HR	Fischer et al. (2007)

Each analysis method in the catalog is classified by dimension (e.g., quantitative or functional), type of the analysis (e.g., performance, cost, availability), and TOGAF Architecture Domain that can be Business Architecture (BA), Data Architecture (DA), Application Architecture (AA), and Technology Architecture (TA). In addition, based on ArchiMate, each analysis method has a unified and well-defined documentation. The documentation of analysis methods provides the required information for understanding the purpose, requirements, mode of use, and results. The documentation also aims to reuse analysis methods due to they are applicable to any ArchiMate model. The documentation contains: (1) id, which is a code for identifying the analysis method that includes three letters indicating the dimension, type, and a consecutive number of three digits (e.g., QAV004 indicating that the analysis method is the fourth that belongs to the dimension quantitative and the type availability); (2) name; (3) ArchiMate layer, which indicates the layer involved in the analysis method (e.g., Business, Application, Infrastructure, Motivation); (4) description; (5) elements, which informs all ArchiMate element types that are used in the analysis method (e.g., BusinessProcess, ApplicationComponent, Device); (6) relations, which informs the required ArchiMate relations between elements used in the analysis method (e.g., UsedBy, Realization, Flow); (7) required attributes, which specifies the involved attributes for running the desired analysis method; (8) algorithm, which presents the procedure for extracting information from the model and calculating the correspondent result; and (9) output, which informs the kinds of results that the analysis method provides (e.g., model enriched, report). Table 3 presents the catalog with the quantitative dimension, while Table 4 presents the catalog with the functional dimension.

**Table 3:** Catalog of quantitative automated analysis methods

Type	Id	Domain	Name
Pe	QPR001	TA	Infrastructure services workload (Iacob and Jonkers 2006)
	QPR002	AA	Application services processing and response time (Iacob and Jonkers 2006)
	QPR003	AA	Application services utilization (Iacob and Jonkers 2006)
Op	QOP001	BA, DA, AA	Estimate process efficiency based on resources usage (Davenport and Short 1990)
	QOP002	BA	Estimate process effectiveness (Davenport and Short 1990)
IoC	QIC001	TA	Modify (increase/decrease) Infrastructure resources (Akkary and Driscoll 1998)
	QIC002	TA	Modify (increase/decrease) Network resources (Donoso 2009)
	QIC003	AA, TA	Modify (increase/decrease) threads of a service / application (concurrency) (Menascé and Bennani 2006)
	QIC004	AA	Modify (increase/decrease) quantity of users, license implications (Di Penta et al. 2010)
CP	QCP001	BA, DA, AA, TA	Estimate overall solution architecture storage volumetric (Somasundaram and Shrivastava 2009)
	QCP002	BA, DA, AA, TA	Estimate overall solution architecture network volumetric (Donoso 2009)
	QCP003	BA, DA, AA, TA	Estimate number of servers required to support operation (Schmidt 2006)
Cos	QCT001	BA, DA, AA, TA	Overall architecture Cost (Quartel et al. 2010)
	QCT002	BA, DA, AA, TA	Overall architecture Cost/Benefit (Quartel et al. 2010)
	QCT003	BA, DA, AA, TA	Overall architecture ROI (Quartel et al. 2010)
	QCT004	BA, DA, AA, TA	Overall architecture required portfolio (Quartel et al. 2010)
Av	QAV001	BA, DA, AA, TA	Estimate overall architecture availability (Schmidt 2006)
	QAV002	BA, DA, AA, TA	Estimate overall architecture RTO (Schmidt 2006)
	QAV003	BA, DA, AA, TA	Estimate overall architecture RPO (Schmidt 2006)
	QAV004	AA	Application component availability (Florez et al. 2014)
TO	QTO001	AA	Application service Performance versus Flexibility (Berander et al. 2005)
	QTO002	AA	Application service Performance versus Security (Berander et al. 2005)
	QTO003	AA	Application interface Performance versus Flexibility (Berander et al. 2005)
	QTO004	AA	Application interface Performance versus Security (Berander et al. 2005)
	QTO005	AA	Application component Performance versus Flexibility (Berander et al. 2005)
	QTO006	AA	Application component Performance versus Security (Berander et al. 2005)
	QTO007	DA	Data Access Performance versus Flexibility (Berander et al. 2005)
	QTO008	DA	Data Access Performance versus Security (Berander et al. 2005)
	QTO009	BA, DA, AA, TA	Overall process Performance versus Flexibility (Berander et al. 2005)
	QTO010	BA, DA, AA, TA	Overall process Performance versus Security (Berander et al. 2005)
HR	QHR001	BA	Human Resource workload at business process level (Armistead et al. 1999)
	QHR002	BA	Human Resource capacity requirements at business process level (Armistead et al. 1999)
	QHR003	BA	Business processes participants (Armistead et al. 1999)
	QHR004	BA	Roles by business process or function that have only one accountable actor (Davenport and Short 1990)
	QHR005	BA	Human resources capacity planning (Armistead et al. 1999)

**Table 4:** Catalog of functional automated analysis methods

Type	Id	Domain	Name
IoC	FIC001	BA, DA, AA, TA	Impact of removing elements (Sunkle et al. 2013)
	FIC002	BA, DA, AA, TA	Impact of removing a relation between two elements (Sunkle et al. 2013)
Al	FAG001	BA, AA	Business-Application Alignment (The Open Group 2012)
	FAG002	BA, TA	Business-Technology Alignment (The Open Group 2012)
	FAG003	AA, TA	Application-Technology Alignment (The Open Group 2012)
Coh	FCH001	BA	Every business active structure has at least one direct/derived assignment (The Open Group 2012)
	FCH002	BA	Every business process realizes as least one business service (The Open Group 2012)
	FCH003	AA, TA	Every application component uses infrastructure service, node, or device (The Open Group 2012)
	FCH004	BA, AA	Every application service is used at least in one business process (The Open Group 2012)
Cor	FCO001	DA	Data security compliance at transport level (Guadamuz 2001)
	FCO002	DA	Data security compliance at persistence level (Guadamuz 2001)
	FCO003	BA	Business layer single point of fail (Lam 2002)
	FCO004	AA	Application layer single point of fail (Schmidt 2006)
	FCO005	TA	Technology layer single point of fail (Schmidt 2006)
	FCO006	BA, DA, AA, TA	Overall architecture single point of fail (Schmidt 2006)
	FCO007	AA	Integration protocols compatibility at application service level (The Open Group 2012)
	FCO008	TA	Communication and transport protocols compatibility at technology level (Donoso 2009)
	FCO009	TA	Requirement compliance at infrastructure level (Florez et al. 2014)
Con	FCF001	BA, DA, AA, TA	Metamodel conformance (Gómez et al. 2014)
	FCF002	DA	Reference architecture conformance at Data/Information level (Angelov et al. 2009)
	FCF003	AA	Reference architecture conformance at Application level (Angelov et al. 2009)
	FCF004	AA	Reference architecture conformance at Application Integration level (Angelov et al. 2009)
	FCF005	TA	Reference architecture conformance at Technology level (Angelov et al. 2009)
Ga	FGP001	BA	Business process Gap Analysis (The Open Group 2011)
	FGP002	DA	Data/Information Architecture GAP Analysis (The Open Group 2011)
	FGP003	AA	Application Architecture GAP Analysis (The Open Group 2011)
	FGP004	AA	Application-Integration Architecture GAP Analysis (The Open Group 2011)
	FGP005	TA	Technology Architecture GAP Analysis (The Open Group 2011)
GS	FGR001	BA, DA, AA, TA	Circular reference (Bollobás 1982)
	FGR002	BA, DA, AA, TA	Derived associations (The Open Group 2012)
	FGR003	BA, DA, AA, TA	Element depth by typed relations (The Open Group 2012)
	FGR004	BA, DA, AA, TA	Minimum spanning tree (Bollobás 1982)
	FGR005	BA, DA, AA, TA	Shortest path by typed relations (Bollobás 1982)
	FGR006	BA, DA, AA, TA	Element dependency (Bollobás 1982)
Cou	FCN001	BA, DA, AA, TA	Counting of elements by types (The Open Group 2012)
	FCN002	BA, DA, AA, TA	Counting of relationships by types (The Open Group 2012)
Pr	FPR001	DA, AA	Data/Information versus Application (Angelov et al. 2009)
	FPR002	BA, DA	Data/Information versus Process (Angelov et al. 2009)
	FPR003	BA	Process responsibility assignment (Kofman et al. 2009)
HR	FHR001	BA	Business layer passive elements RACI matrix (Fischer et al. 2007)
	FHR002	AA	Application layer passive elements RACI matrix (Fischer et al. 2007)
	FHR003	TA	Technology layer passive elements RACI matrix (Fischer et al. 2007)

We also have classified all analysis methods in the Zachman Framework for Enterprise Architecture (Zachman 2006) in order to identify the audience perspectives and classification names of each method. Table 5 presents the resulting classification. This classification was done by identifying the analysis methods that satisfies each intersection of the framework, where intersections combine the primitive interrogatives of the framework (*What*, *How*, *When*, *Who*, *Where*, and *Why*) with the transformations also identified in the framework (*Identification* (Scope Context), *Definition* (Business Concepts), *Representation* (System Logic), *Specification* (Technology Physics), *Configuration* (Tool Components), and *Implementation* (Operation Instances)). For instance, the method *QIC002: Modify* (*increase*/*Decrease*) *Network resources* was included in two intersections of the framework: the first one is the intersection of the primitive *Where* and the transformation *Specification* (Technology Physics), and the second one is the intersection of the primitive *Where* and the transformation *Configuration* (Tool Components). The method was placed in these intersections because (a) it is intended to adjust the amount of infrastructure resources required for the right operation of IT systems in an organization and (b) the cells defines the IT distribution specification and configuration.

**Table 5:** Analysis methods classification in the Zachman Framework

	What	How	Where	Who	When	Why	
Executive	QCT001 QCT002	QCT003 QCT004	FCO003 FCO006	QHR005	QTO009 FGP001	QTO010	Scope Context
Business management	QAV001 FHC001 FCF002 FPR001	QAV002 QAV003 FHC002 FPR002	FAG001 FAG002 FCH004 FCO003	QOP002 QHR001 QHR003 QHR004 FHR001	QOP001 QTO007 QTO009	QTO008 QTO010 QHR002 FPR003	Business Concepts
Architect	QCP003 QAV001 FIC001 FIC002 FCF004 FGR00*	QCP001 QAV002 QAV003	QCP002 FAG001 FAG003 FCO004	QHR001 FCO001 FCO002 FHR002	FGP002	QHR002	System Logic
Engineer	QPR001 QAV004 FCF003 FCF005 FCN001 FCN002 FGR00*	QPR003 QIC001	QIC002 FAG002 FAG003 FCH003 FCO005 FCO007	QIC004 FHR003	QPR002 QTO001 QTO003 QTP005 FGP003 FGP004	QIC003 QTO002 QTO004 QTO006 FCO009	Tech. Physics
Technician	QPR001 QAV004	QPR003 QIC001	QIC002 FCO008	QIC004 FHR003	QPR002 FGP005	QIC003 FCO009	Tool Comp.
Enterprise	FCF001 QCT001 QCT002	QCT003 QCT004	FCO006	QHR001	FGP001 QTO009	QTO010	Op. Instances
	Invent. Sets	Process Flows	Distrib. Network	Respons. Assignm.	Timing Cycles	Motivat. Intention	

* Analysis methods FGR001, FGR002, FGR003, FGR004, FGR005, and FGR006

In this paper, we just illustrate the quantitative analysis methods: QHR003 and QAV004 and the functional analysis methods: FIC001 and FCO009. However, all information of the catalog is available in http://catalog.virtual.uniandes.edu.co. We have selected these analysis methods in order to illustrate the following situations: (a) results that require specific attributes and enrich the model including new attributes; (b) results that provide facts, but require specific attributes; and (c) results that require attributes and enrich the model including new relations. All analysis methods have a unified documentation shown in the illustrated methods.

### Quantitative analysis method: business processes participants

Table 6 presents the documentation of the analysis method *QHR003: Business Processes Participants*, which enumerates the business actors and business roles associated with all business processes. This association can be direct or indirect. On the one hand, actors or roles are directly associated with one process, when there is an Assignment relation from the actor or role to the process. On the other hand, actors or roles are indirectly associated with one process, when there are business interactions, business collaborations, or business functions between the actors or roles and the process. This method illustrates (a) the need of the correspondent relations between business elements and (b) the reporting of the analysis method results with specific details.

**Table 6:** Automated analysis method: *QHR003*

ID: QHR003
Name: Business Processes Participants
Dimension: Quantitative
Type: Human Resources (HR)
Description: This method reports all business processes relating their business actors and business roles. The relation between them can be indirect through business collaboration or business interaction. In addition, one business process can be composed by several business processes. In this case, the actor or role are related with both the container and the contained business processes
ArchiMate Layers: Business
Elements: BusinessProcess, BusinessCollaboration, BusinessActor, BusinessRole
Required Relations: Aggregation from BusinessCollaboration to BusinessActor or BusinessRole; Assignment from BusinessCollaboration, BusinessActor, or BusinessRole to BusinessProcess or BusinessInteraction; Flow from or to BusinessInteraction and BusinessProcess
Required Attributes: None
Output: Report with the following information: (a) business process name; (b) participant name; (c) participant type (e.g., BusinessActor or BusinessRole); and (d) details that specify the intermediate elements between participants and processes (e.g., BusinessInteraction or BusinessCollaboration)

Algorithm 1 calculates the results of the analysis method QHR003, where *BP* = *BusinessProcess*, *BA* = *BusinessActor*, *BR* = *BusinessRole*, *BC* = *BusinessCollaboration*, and *BI* = *BusinessInteraction*. The algorithm iterates all business processes. For each business process, the algorithm gets the relations and verifies whether each relation targets one business actor or business role. If true, the business actor or business role is collected; however, if false, the algorithm verifies whether the consulted relation targets one business collaboration or business interaction. If true, the algorithm gets the relations of the gathered business collaboration or business interaction and verifies whether each relations targets one business actor or business role, for collecting them as well. 
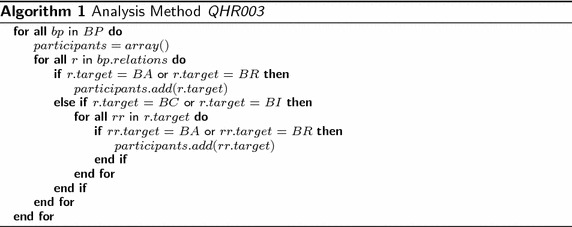


**Fig. 1:**
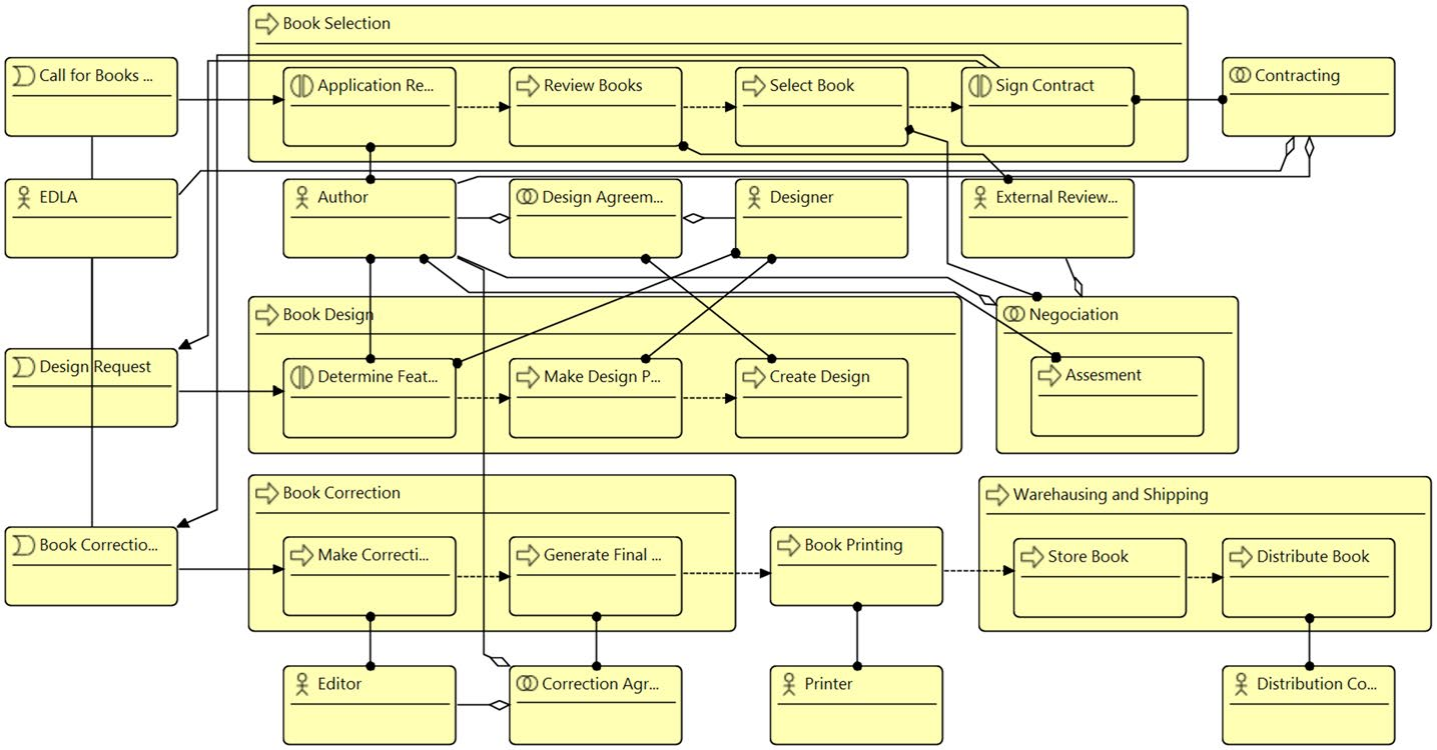
Fragment of business process view of the publisher scenario. Analysis method QHR003 reports the actors or roles related with processes

Figure 1 presents one fragment of the business process view of the enterprise model of the publisher scenario. It includes several business processes, where some of them are related with other business processes through a Composition relation (i.e., container) or Flow relation. The model also includes business interactions related with business processes through Flow relation as well. Business collaborations are related with business processes and business interactions trough Assignment relations. Finally, business actors and business roles are related with business processes, business interactions, or business collaborations through Assignment relations. The analysis method *Business Process Participants* has been run using this model.

**Fig. 2:**
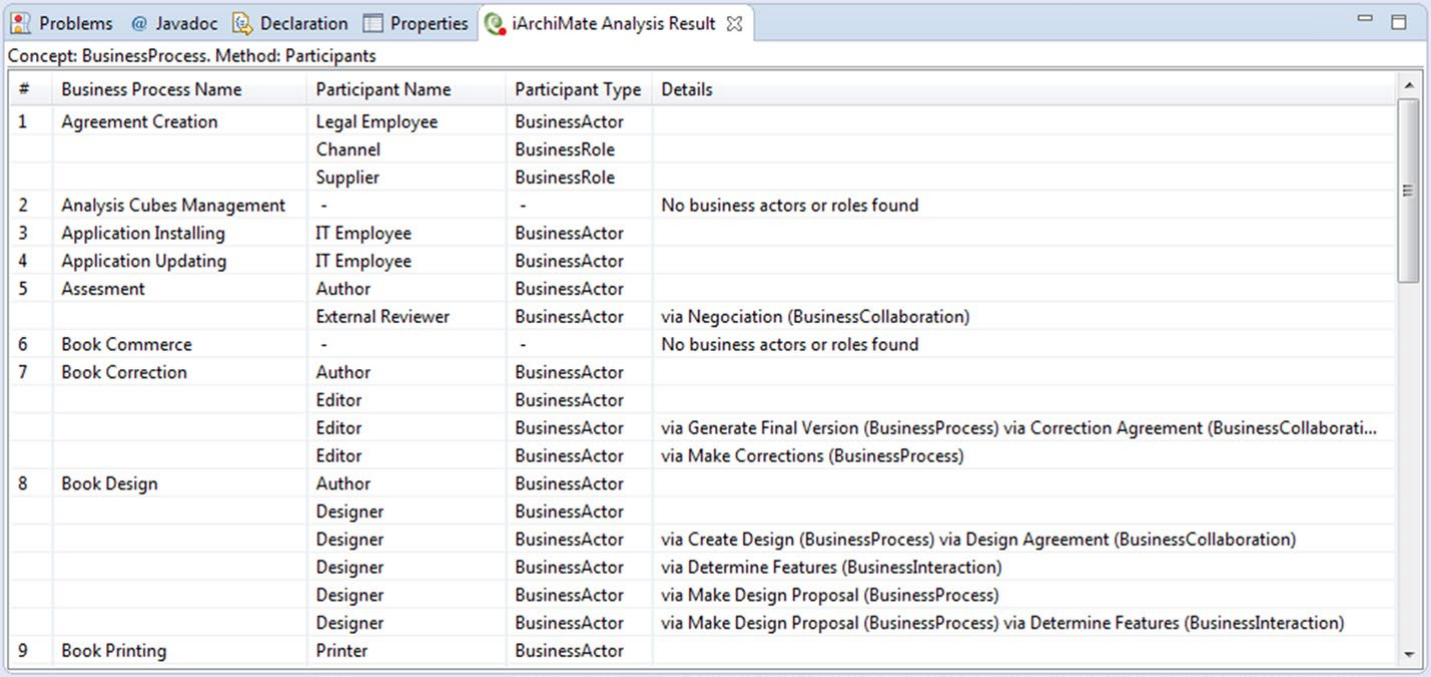
Analysis results. Report of analysis method: QHR003

Figure 2 presents one part of the results of the analysis method. The result is a report that includes all business processes with the correspondent associated business actors or business roles. In addition, the report includes details when the relation between the business process and the business actor or business role is indirect; thus, the details informs the business elements involved in the relation.

### Quantitative analysis method: application component availability

Table 7 presents the quantitative automated analysis method *QAV004: Application Component Availability*. This analysis method is intended to calculate the availability of all application components based on the related infrastructure elements. This method illustrates the need of the required attribute availability in Device, the reporting of the analysis method results, and the enrichment of the model with new attributes (availability in SystemSoftware, InfrastructureService, and ApplicationComponent).

**Table 7:** Automated analysis method: *QAV004*

ID: QAV004
Name: Application Component Availability
Dimension: Quantitative
Type: Availability
Description: This availability analysis method aims to calculate the availability of all application components based on the availability of the devices, system software, or infrastructure services that support them. The availability value of system software and infrastructure services is also calculated
ArchiMate Layers: Application, Infrastructure
Elements: ApplicationComponent, SystemSoftware, InfrastructureService, Device
Required Relations: Aggregation from Device to SystemSoftware; Realization from Device to InfrastructureService; UsedBy from Device, SystemSoftware, and InfrastructureService to ApplicationComponent
Required Attributes: availability in all Device
Output: (1) Enriched model with the attribute availability in all application components, system software, and infrastructure services. (2) Report with the following information: (a) element type; (b) element name; (c) availability value; (d) associated elements, which were used to calculate the availability; and (e) action (e.g., availability created or availability updated)

Algorithm 2 calculates the results of the analysis method QAV004, where *IE* = *Infrastructure Element* such as *Device*, *SystemSoftware*, or *InfrastructureService*; *AC* = *ApplicationComponent*; *A* = *Association*; *C* = *Composition*; R = *Realization*; *UB* = *UsedBy*. The algorithm iterates all infrastructure elements (i.e., devices, infrastructure services, and system software). For each infrastructure element, the algorithm gets the relations and verifies whether each relation targets one application component, infrastructure service, or system software with the correspondent relation (i.e., used by, realization, or aggregation/composition respectively). If true, the availability of the target element is multiplied by the availability of the infrastructure element.
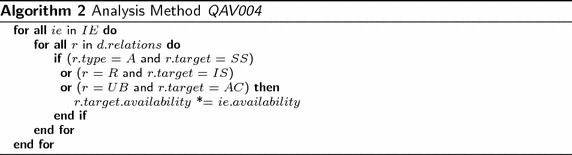


**Fig. 3:**
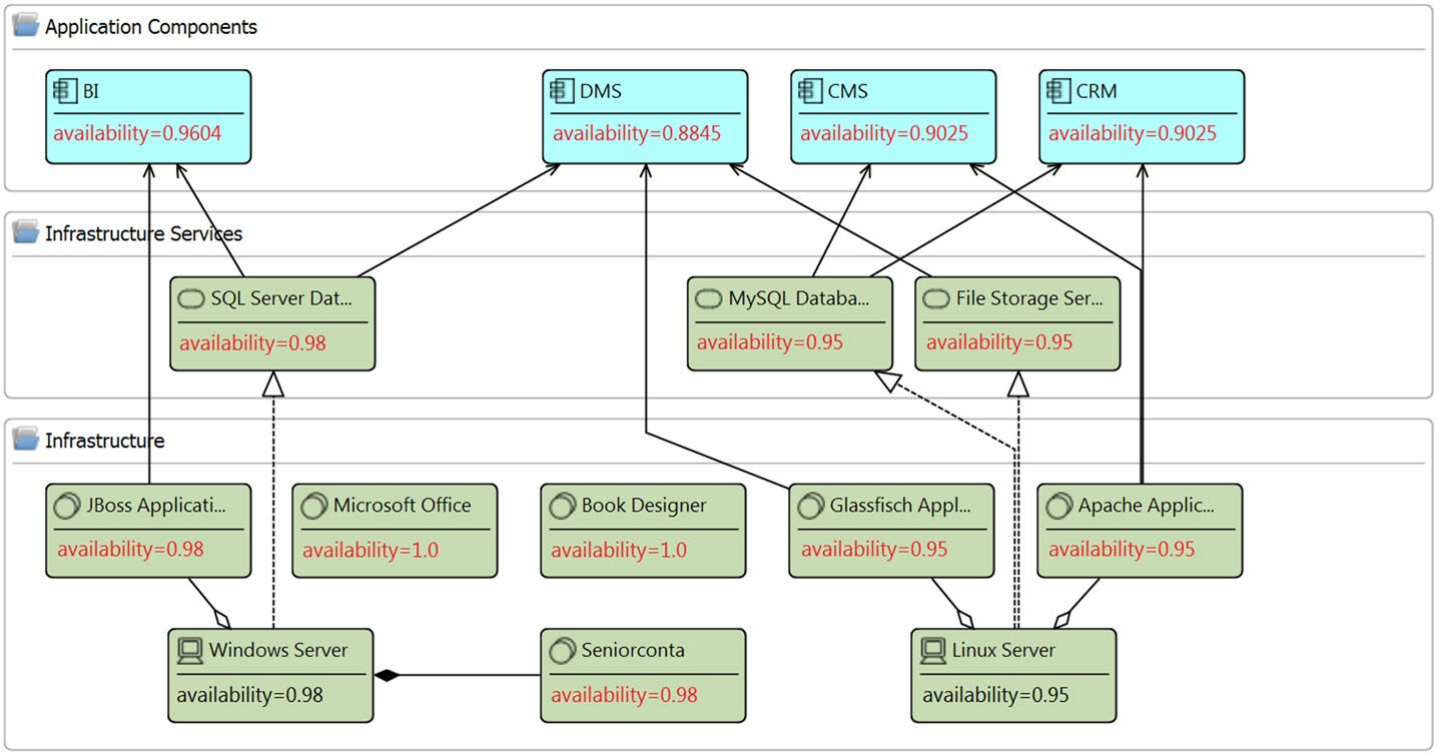
Fragment of layered view of the publisher scenario. Attributes in *red text* have been created by the analysis method QAV004

**Fig. 4:**
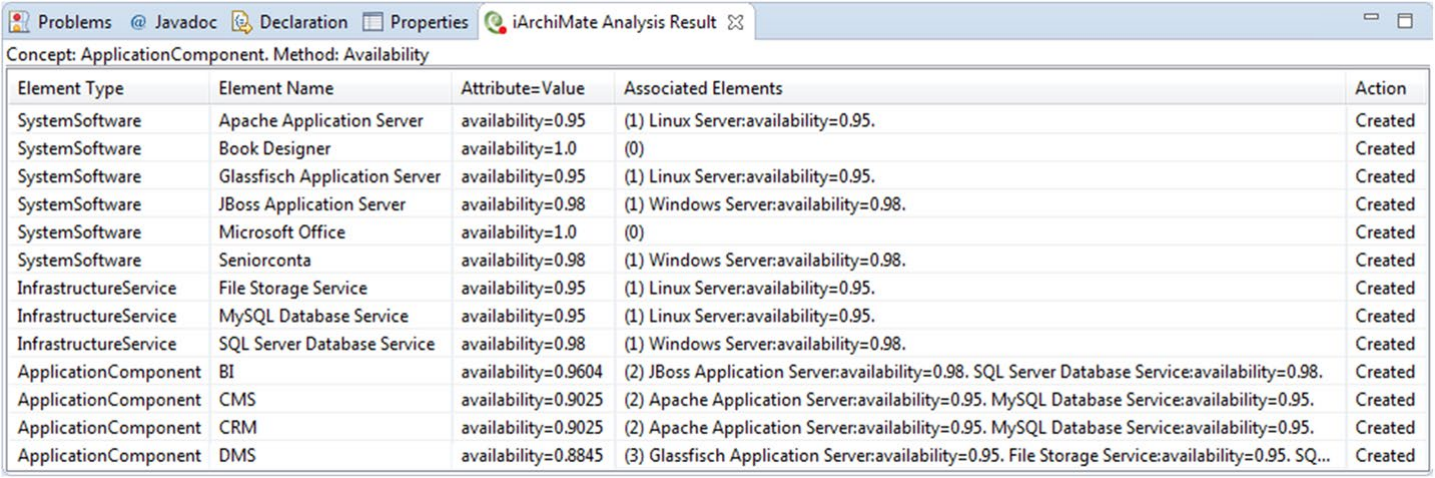
Analysis results. Report of analysis method: QAV004

Figure 3 presents one fragment of the layered view of the enterprise model of the publisher scenario that includes elements from the application and infrastructure layers. It has the devices *Windows Server* and *Linux Server*, which aggregates some system software and realizes some infrastructure services. In addition, the model contains the application components *BI*, *DMS*, *CMS*, and *CRM*. Some infrastructure elements are used by the mentioned application components. This model has been used for running the analysis method *Application Component Availability*; thus, the attribute availability in the devices *Windows Server* and *Linux Server* is mandatory. In this model the attribute availability (shown in red) in all system software, infrastructure services, and application components is introduced by running the analysis method. Figure 4 reports the results of the analysis method. Before calculating the availability in the application components, the availability of system software and infrastructure services is required to be calculated. The report presents the calculated availability value for the involved elements. For instance, the report indicates that the availability of the ApplicationComponent*CRM* is *0.9025* and it was calculated based on the SystemSoftware*Apache Application Server* and the InfrastructureService*MySQL Database Service*. For this method, the ArchiMate metamodel has been extended including the attribute availability in the types Device, SystemSoftware, InfrastructureService, and ApplicationComponent.

### Functional analysis method: impact of removing elements

Table 8 presents the functional automated analysis method *FIC001: Impact of Removing Elements*, which is intended to assess the elements impacted when one element of the model is removed. This method illustrates the need of the attribute remove in any element and the reporting of the analysis method results.

**Table 8:** Automated Analysis Method: *FIC001*

ID: FIC001
Name: Impact of Removing Elements
Dimension: Functional
Type: Impact of change
Description: This analysis method aims to assess the impact of removing one element from the model by means of the use of the attribute remove in the element in order to simulate a logical removing process. The attribute remove is optional; then, the analysis method will analyze all elements that include the attribute remove with the value *true*
ArchiMate Layers: All Layers
Elements: All elements in the model
Required Relations: Any ArchiMate relation with any source and target element
Required Attributes: remove in desired elements
Output: Report with the following information: (a) element name; (b) element type; (c) impacted elements name; (d) impacted elements type; and (e) details that specifies the intermediate elements between the removing element and the impacted elements

Algorithm 3 calculates the results of the analysis method FIC001, where *E* = *Elements*. The algorithm iterates all elements in the model. For those elements that include the attribute remove, the algorithm collects their target elements. Later, the algorithm iterates the collected elements and collects again their targets. Thus, the algorithm recursively can collect all direct and indirect dependent elements. The algorithm does not modify the model; thus, its output is a report that specifies all direct dependent elements and all indirect dependent elements with their correspondent trace of intermediate dependent elements.
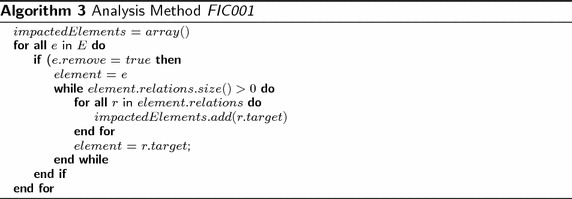


**Fig. 5:**
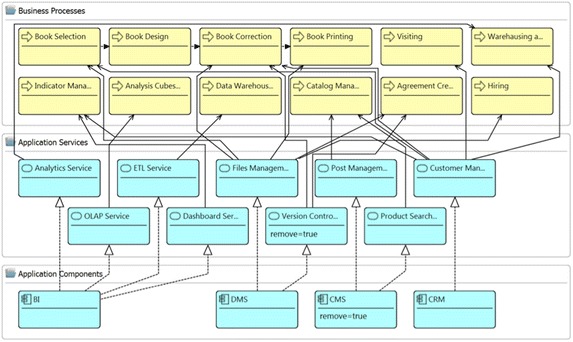
Fragment of layered view of the publisher scenario. Analysis method FIC001 checks the value of the attribute remove

**Fig. 6:**
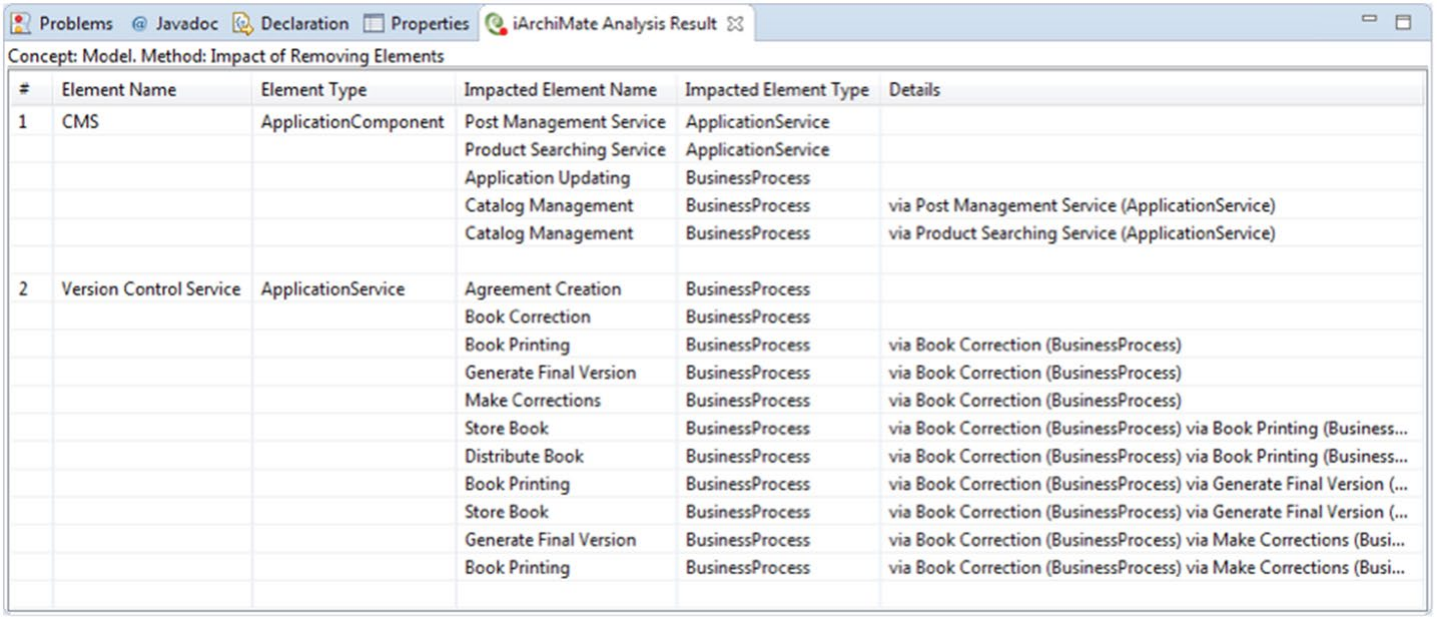
Analysis results. Report of analysis method: FIC001

Figure 5 presents the other fragment of the layered view (presented in Fig. 3) of the enterprise model of the publisher scenario that includes elements from the business and application layers. It contains application components, which realizes several application services that are used by some business processes. The attribute remove with the value *true* has been introduced in the application component *CMS* and in the application service *Version Control Service*. This fragment of the model has been used for running the analysis method Impact of Removing Elements. Figure 6 reports the results of the analysis method. The report presents the list of the impacted elements that depends directly or indirectly on the elements *CMS* or *Version Control Service*. When the impacted element has an indirect dependency, the report indicates the intermediate elements.

### Functional analysis method: requirement compliance at infrastructure level

Table 9 presents the functional automated analysis method *FCO009: Requirement Compliance at Infrastructure Level*. This analysis method creates relations between requirements and IT elements, when the IT element complies the requirement condition. This method illustrates the need of required attributes, the reporting of the analysis method results, and the enrichment of the model with new relations (Association between Requirement and the correspondent infrastructure elements).

**Table 9:** Automated analysis method: *FCO009*

ID: FCO009
Name: Requirement Compliance at Infrastructure Level
Dimension: Functional
Type: Correctness
Description: This analysis method aims to create an association relation between requirements and infrastructure elements such as devices, infrastructures services, or system software, when the element does not comply with the condition specified in requirements. The condition is given by the attributes: (a) *conditionAttribute*, (b) conditionOperation, (c) conditionValue, and (d) targetElements
ArchiMate Layers: Motivation, Infrastructure
Elements: Requirement, SystemSoftware, Device, InfrastructureService
Required Relations: None
Required Attributes: conditionAttribute, condition Value, conditionOperation, and targetElements in Requirement. $\ll$conditionAttribute$\gg$ (e.g., availability) in all $\ll$targetElements$\gg$ (e.g., Device)
Output: Report with the following information: (a) requirement name; (b) requirement attributes; (c) associated elements; (d) associated elements type; (e) condition attribute with the correspondent value, and (f) action (e.g., relation created)

Algorithm 4 calculates the results of the analysis method FIC001, where *R* = *Requirement*. The algorithm iterates the requirements of the model and gets the values of the required attributes conditionAttribute, conditionOperation, conditionValue, and targetElements. For each requirement, the algorithm iterates all elements specified in targetElements. For each of those elements, the algorithm gets the value of the attribute specified in conditionAttribute and makes the comparison specified in conditionOperation with the value specified in conditionValue. If the comparison is *true*, the Association relation is created.
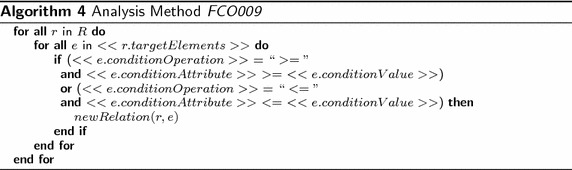


**Fig. 7:**
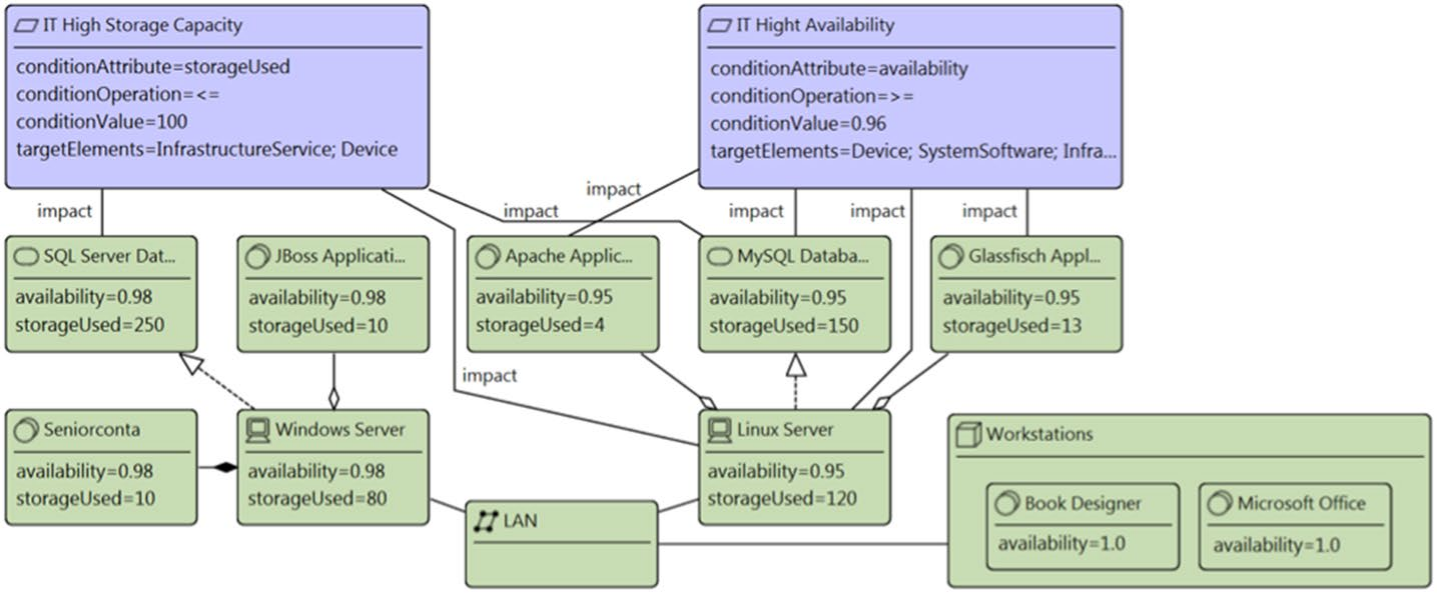
Infrastructure technology view of the publisher scenario. Relations impact have been created by the analysis method FOC009

**Fig. 8:**
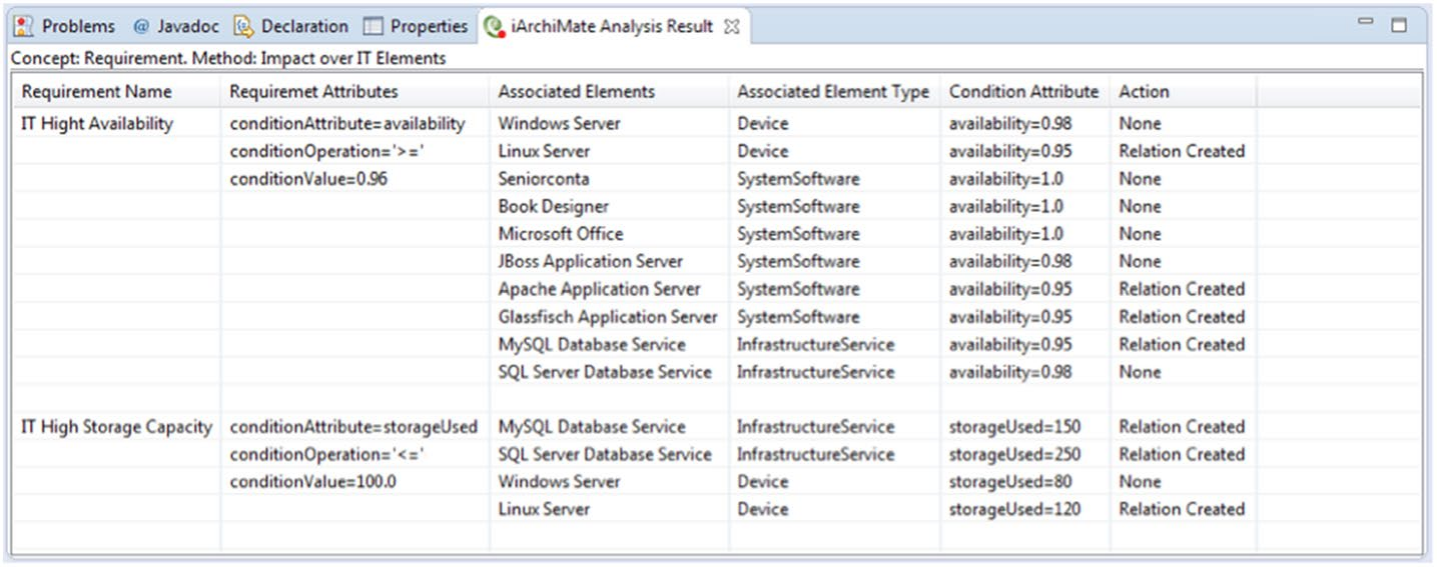
Analysis results. Report of analysis method: FOC009

Figure 7 presents the infrastructure technology view of the enterprise model of the publisher scenario with two requirements. It contains devices, which realizes infrastructure services and aggregates system software. In addition, the model has the requirements: (1) *IT High Availability* with the attributes: targetElements = “*Device, SystemSoftware, InfrastructureService*”, conditionAttribute = “*availability*”, conditionOperation = “>=”, and conditionValue = “*0.96*”; and (2) *IT High Storage Capacity* with the attributes: targetElements = “*Device, InfrastructureService*”, conditionAttribute = “*storageUsed*”, conditionOperation = “<=”, and conditionValue = “*100*”. Finally, the model has relations called impact from the requirements to some infrastructure elements. Those relations were created by the analysis method, when the attribute (e.g., availability, storageUsed) value in the element does not comply with the condition established in the requirements. Figure 8 reports the results of the analysis method. The report presents the list of the requirements with the condition attributes and the infrastructure elements specifying the relations created between requirements and elements.

## A tool for supporting automated analysis

We built *iArchiMate* (see http://iarchimate.virtual.uniandes.edu.co), which is an enterprise modeling and analysis tool (Florez et al. 2014). The tool’s core is a graphical editor based on Eclipse Modeling Framework Project (EMF) and Graphical Modeling Framework Project (GMF). *iArchiMate* is capable of validating the model providing assistance to the user, in order to determine if the model fulfils the required information for running the desired automated analysis method (e.g., attribute availability in all Device for running the method QAV004). This validation is supported by Epsilon Validation Language (EVL); thus, when the desired analysis method is selected, iArchiMate generates an EVL script, for validating the model.

**Fig. 9:**
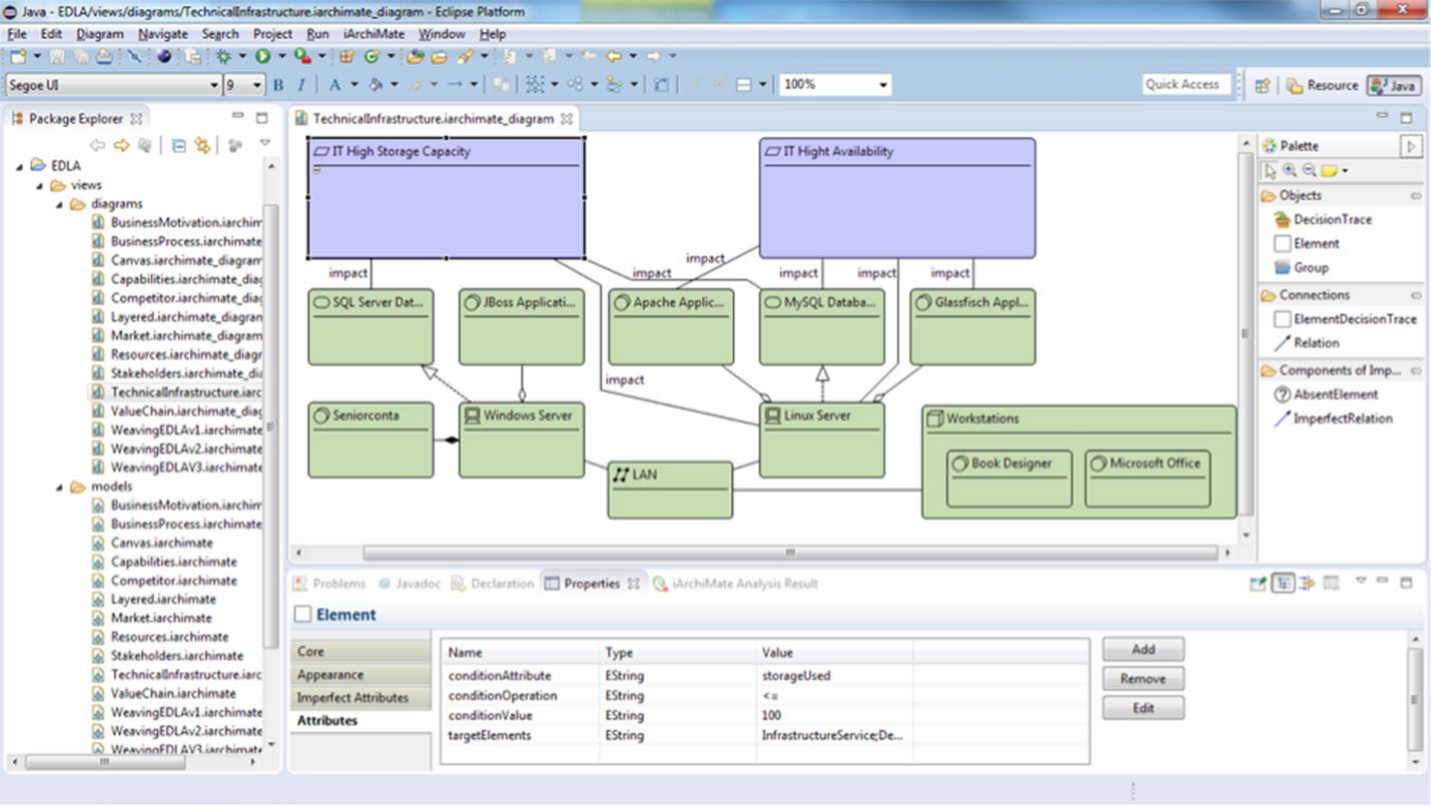
Screenshot of iArchiMate. *iArchiMate* has in the right side a palette for drawing elements and relations and in the bottom side the *Properties* view and *Analysis results* view

Moreover, iArchiMate allows importing models from other modeling tools such as Archi (see http://www.archimatetool.com). Then, *iArchiMate* imports the model leaving it ready to include the additional information that analyses require. Figure 9 presents a screenshot of *iArchiMate* and shows the *Technical Infrastructure View* of the enterprise model of the publisher scenario. In the left side, the package explorer allows browsing the views of the model. In right side of the graphical editor, there is the palette, which includes components for creating ArchiMate models. The tool allows selecting the ArchiMate type of elements (e.g., BusinessProcess, ApplicationComponent) and relations (e.g., UsedBy, Flow) through one attribute called typeName. At the bottom of *iArchiMate*, there is the properties view, which includes a tab for displaying the attributes of the selected element or relation and the *iArchiMate Analysis Result* view. In addition, there is a menu, for selecting and performing analysis methods.

### Automated analysis methods

Although *iArchiMate* can be used to create ArchiMate models, its real value is in supporting the execution of automated analysis methods. For achieving this, the modeler must select the desired analysis method, from a list of the currently deployed ones. With this information, *iArchiMate* generates the necessary EVL scripts, for validating the existence of all mandatory information that the method requires. After the modeling phase is finished and the validation process is successful, the analyst can run any of the enabled analysis methods and its results are collected.

**Fig. 10:**
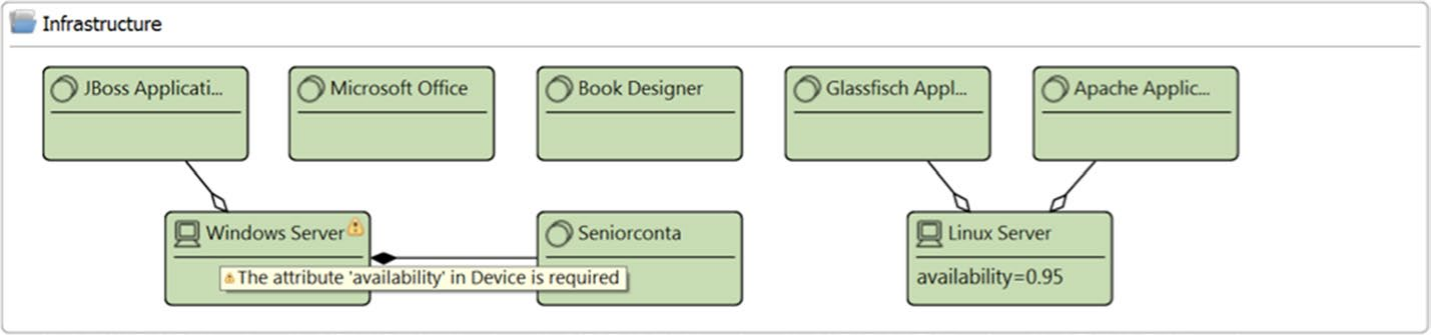
Validation result. The validation provides information regarding the mandatory attributes required for a specific analysis method

If some mandatory information is not available, the method cannot be run and the problem is highlighted. The validation engine produces a warning laying it up on top of the diagram and listing it in the Eclipse *Problems* view. For instance, the analysis method *Application Component Availability*, illustrated in the previous section, requires the attribute availability in all Device; then, after selecting this analysis method, the validation engine verifies this requirement. Figure 10 presents the elements of the infrastructure group presented in Fig. 3, which corresponds to the layered view of the publisher scenario. For illustrating the validation result, the attribute availability in the Device*Windows Server* has been deleted from the model; consequently, a warning appears informing the problem.

### Results of analysis

The results of an analysis method can have two natures. On the one hand, there are results, which are external to the model and need to be exported or visualized in a separated way. In *iArchiMate*, these kinds of results are visualized in a view called *iArchiMate Analysis Results*, which can display simple values as well as more complex tables. On the other hand, there are analysis methods that can enrich the model. These methods may register their results as valued attributes of elements or relations, as new relations, or even as new elements. New relations and elements are highlighted in the model. Executing the automated analysis methods is not necessarily the last step in the process because new analysis methods can be selected and run at any time in order to provide additional results.

### iArchiMate architecture

**Fig. 11:**
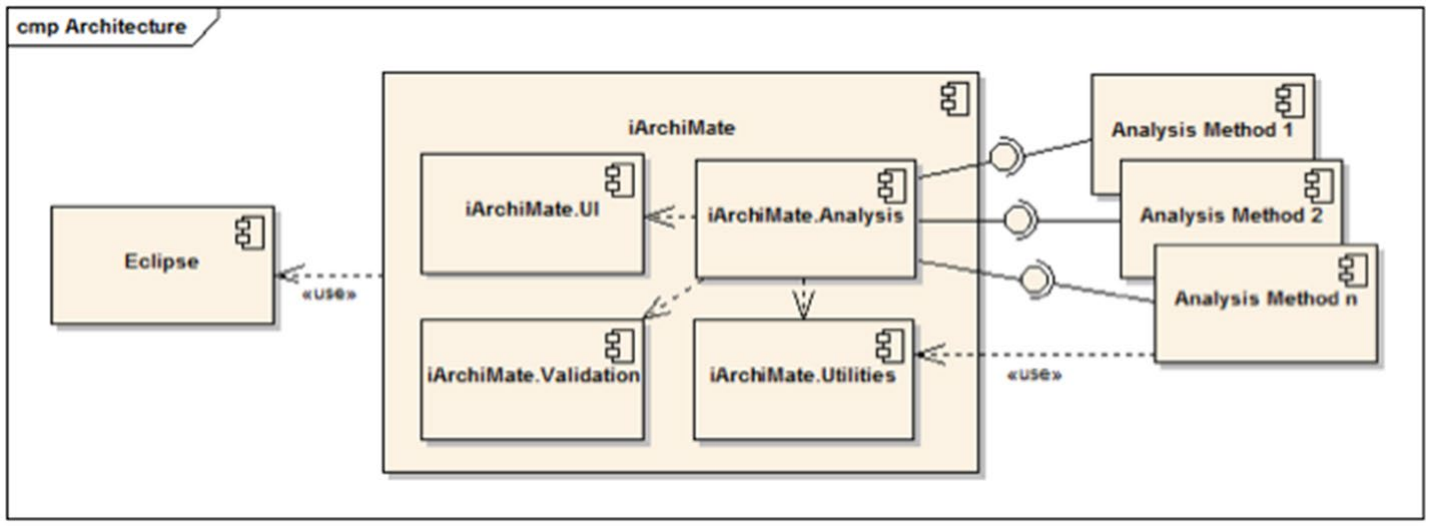
*iArchiMate* architecture. The architecture allows including further analysis methods

*iArchiMate* architecture (see Fig. 11) is extensible, supporting the implementation of new analysis methods, when the catalog is extended. This can be done because the architecture has one analysis component, which is the analysis core. The analysis component uses (1) the user interface component, which provides wizards, message boxes, and the analysis results view; (2) the validation component, which provides the mechanisms for creating the EVL scripts and the validation engine; and (3) the utilities components, which provides services for manipulating the model based on EMF. The creation of a new analysis method implies creating a component that uses the services exposed by the analysis component.

## Conclusions

Enterprise modeling and enterprise modeling analysis are becoming increasingly important in many fields. There are some approaches that offer different alternatives for analyzing enterprise models. However, these approaches are focused on specific kinds of analyses; then, they usually provide a limited number of analysis methods. Based on this situation, it is unlikely to be able to cover all analysis options.

In this paper we have presented the work of compilation, classification, structuring, characterization, and unification of automated analysis methods for enterprise models. We collected these analysis methods in one catalog, which aims to offer a guide for supporting enterprise analysts in analyses processes through the description of 78 analysis methods sorted in 2 dimensions (quantitative and functional) and 16 analysis types.

The paper also presented our tool called *iArchiMate* in which we have implemented the analysis methods presented in the catalog, for ArchiMate models, and offers a framework for the development of additional methods. By using *iArchiMate* it is possible to create or import ArchiMate models; to validate models based on the analysis method that is intended to be performed; and to run the selected analysis methods, where the results can be enrich the model.
